# Decoding Diabetes Biomarkers and Related Molecular Mechanisms by Using Machine Learning, Text Mining, and Gene Expression Analysis

**DOI:** 10.3390/ijerph192113890

**Published:** 2022-10-26

**Authors:** Amira M. Elsherbini, Alsamman M. Alsamman, Nehal M. Elsherbiny, Mohamed El-Sherbiny, Rehab Ahmed, Hasnaa Ali Ebrahim, Joaira Bakkach

**Affiliations:** 1 Department of Oral Biology, Faculty of Dentistry, Mansoura University, Mansoura 35116, Egypt; 2 Agricultural Genetic Engineering Research Institute, Agricultural Research Center, Giza 12619, Egypt; 3 Department of Pharmaceutical Chemistry, Faculty of Pharmacy, University of Tabuk, Tabuk 71491, Saudi Arabia; 4 Department of Biochemistry, Faculty of Pharmacy, Mansoura University, Mansoura 35116, Egypt; 5 Department of Basic Medical Sciences, College of Medicine, AlMaarefa University, Riyadh 71666, Saudi Arabia; 6 Department of Anatomy, Mansoura Faculty of Medicine, Mansoura University, Mansoura 35116, Egypt; 7 Department of Natural Products and Alternative Medicine, Faculty of Pharmacy, University of Tabuk, Tabuk 71491, Saudi Arabia; 8 Department of Pharmaceutics, Faculty of Pharmacy, University of Khartoum, Khartoum 11111, Sudan; 9 Department of Basic Medical Sciences, College of Medicine, Princess Nourah bint Abdulrahman University, Riyadh 11671, Saudi Arabia; 10 Biomedical Genomics and Oncogenetics Research Laboratory, Faculty of Sciences and Techniques of Tangier, Abdelmalek Essaâdi University Morocco, Tétouan 93000, Morocco

**Keywords:** diabetes, text mining, gene expression, bioinformatics, protein–protein interaction network

## Abstract

The molecular basis of diabetes mellitus is yet to be fully elucidated. We aimed to identify the most frequently reported and differential expressed genes (DEGs) in diabetes by using bioinformatics approaches. Text mining was used to screen 40,225 article abstracts from diabetes literature. These studies highlighted 5939 diabetes-related genes spread across 22 human chromosomes, with 112 genes mentioned in more than 50 studies. Among these genes, *HNF4A*, *PPARA*, *VEGFA*, *TCF7L2*, *HLA-DRB1*, *PPARG*, *NOS3*, *KCNJ11*, *PRKAA2*, and *HNF1A* were mentioned in more than 200 articles. These genes are correlated with the regulation of glycogen and polysaccharide, adipogenesis, AGE/RAGE, and macrophage differentiation. Three datasets (44 patients and 57 controls) were subjected to gene expression analysis. The analysis revealed 135 significant DEGs, of which *CEACAM6*, *ENPP4*, *HDAC5*, *HPCAL1*, *PARVG*, *STYXL1*, *VPS28*, *ZBTB33*, *ZFP37* and *CCDC58* were the top 10 DEGs. These genes were enriched in aerobic respiration, T-cell antigen receptor pathway, tricarboxylic acid metabolic process, vitamin D receptor pathway, toll-like receptor signaling, and endoplasmic reticulum (ER) unfolded protein response. The results of text mining and gene expression analyses used as attribute values for machine learning (ML) analysis. The decision tree, extra-tree regressor and random forest algorithms were used in ML analysis to identify unique markers that could be used as diabetes diagnosis tools. These algorithms produced prediction models with accuracy ranges from 0.6364 to 0.88 and overall confidence interval (CI) of 95%. There were 39 biomarkers that could distinguish diabetic and non-diabetic patients, 12 of which were repeated multiple times. The majority of these genes are associated with stress response, signalling regulation, locomotion, cell motility, growth, and muscle adaptation. Machine learning algorithms highlighted the use of the *HLA-DQB1* gene as a biomarker for diabetes early detection. Our data mining and gene expression analysis have provided useful information about potential biomarkers in diabetes.

## 1. Introduction

Diabetes mellitus is a common chronic and debilitating disease. It refers to a set of metabolic disorders that are characterized by chronic elevation of blood glucose, which occurs because of imperfections in insulin action and/or secretion [1]. Diabetes prevalence has increased significantly as a result of changes in sedentary lifestyle, increased fat intake, overweight and obesity, and an ageing population [2]. As reported by the International Diabetes Federation (IDF) in 2019 [3], diabetes affects approximately 463 million people worldwide and is expected to affect 700 million people by 2040.

Due to its associated macro- and microvascular complications that target various body organs, resulting in disability, worsening of life quality and mortality, diabetes is currently imposing a serious burden on health systems worldwide [4] and is considered one of the fastest growing health crises with a massive global economic burden. In this context, the estimated annual expenditure for diabetes was 760 billion USD in 2019 and it is expected to reach 845 billion USD by 2045 [5]. Stopping the spread of the diabetes epidemic in society is therefore critical. This can be accomplished through the development of novel strategies for controlling hyperglycemia and managing diabetes complications, resulting in an improved quality of life. However, a thorough understanding of the disease’s molecular basis is required to achieve this goal.

Differential expression analysis is a powerful tool for identifying disease-related genes. It has been used to study a wide range of human diseases, yielding detailed profiles of up- and downregulated genes [6]. Gene expression data from microarray and whole transcriptome sequencing experiments is now available in massive public databases. These gene expression data-enabled medical research teams validate and re-analyze the data by using a variety of analytical procedures to discover new key factor genes involved in chronic diseases [7]. Furthermore, numerous attempts have been made to connect various data types by using advanced methods in order to build multi-omics data analysis, which could aid in understanding disease biological systems on multiple levels [8].

Various techniques are currently being used to identify diabetes-associated genes and thus gain insights into the disease pathogenesis mechanisms. Wide application of these techniques results in the production a large amount of core slice data. Most of these data are already available in public databases and their re-analysis can provide significant clues for scientific research. Therefore, sophisticated statistical and computational approaches are commonly used to evaluate existing medical knowledge. Due to the massive expansion of medical literature, text mining, and machine learning are two of these approaches that have sparked a lot of interest in the analysis of medical data [9,10]. Text mining involves several steps, including systematic extraction of information from various medical textual resources, visualization, and evaluation [11]. Text mining has been used in medical research to investigate chronic diseases, genetic disorders, and drug discovery [12]. Text mining is used to assess genes linked to chronic disease to better understand their biological function and role in disease manifestation [13].

Machine learning (ML) is the central topic of artificial intelligence technology, which is a rapidly evolving branch that aims to mimic human intelligence by learning from its surroundings [14]. Machine learning is now playing a critical role in the development of learning statistical models capable of assisting healthcare systems [15]. Many supervised and unsupervised ML techniques have been used to identify the most significant genes in gene expression data. These methods are extremely helpful in understanding the structure of gene networks and developing disease risk-prediction models [16]. Several methods for improving the interpretability of ML predictions have been developed, including explainable artificial intelligence (XAI), which suggests relationships between various variables required for outcome prediction [17]. Accordingly, gene expression analyses, data mining, and machine learning were conducted in this study to shed light on the possible controlling genes of diabetes to improve our understanding of the molecular basis of the disease.

## 2. Materials and Methods

### 2.1. Text Mining Analysis

**Figure 1:**
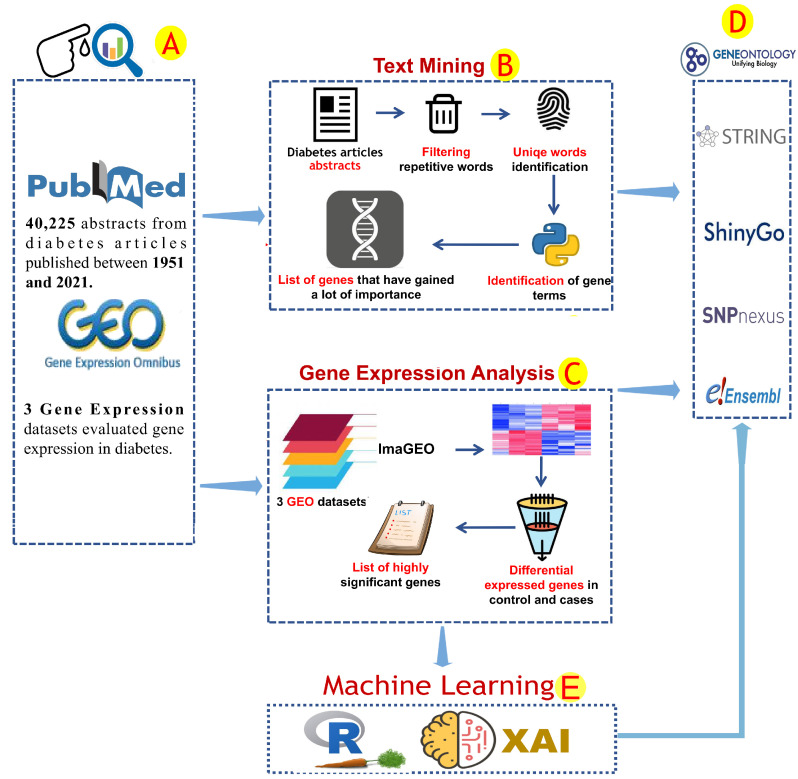
The analytical procedures used in this study. The information used to find genes related to diabetes was obtained from the NCBI and GEO databases (**A**). This data was analyzed by using two different protocols depending on the data type (**B**,**C**). Text mining was used to explore text data that covered some diabetes literature based on gene factors (**B**). Several analytical steps were performed during the text mining analysis, including the removal of repeated words from the article data and the identification of unique gene terms (**B**). ImaGEO software was used to analyze the gene expression data and identify common gene expression patterns. Only significant patterns were reported (fdr-pvalue leq 0.001) (**C**). The gene factors linked to diabetes identified in previous analyses were subjected to gene annotation analysis (**D**). By using gene expression data, machine learning methods such as the R-caret package and Python-XAI were used to identify important genetic biomarkers (**E**).

Text mining is a rich resource for the acquisition of knowledge from the current research literature. However, it requires an elevated level of data filtration and manipulation skills [18]. The available diabetes reports were explored. The National Library of Medicine at the National Institutes of Health (PubMed-NCBI) (https://pubmed.ncbi.nlm.nih.gov/, accessed on 1 October 2020) was used to retrieve all abstracts of scientific articles that reported diabetes-associated genes (Figure 1A). The query of “Diabetes mellitus + gene” was used to download all abstracts of medical articles published from 1951 to 25 February 2021. The text mining analysis included 40,285 abstracts (Figure 1A). Data mining was conducted through the Python programming language. Common English phrases and word redundancy have been removed (Figure 1B). A list of human gene terminology has been prepared by using the human genome hg38, which has been obtained from the NCBI database. Only genes found in more than 50 articles were used for further investigation.

### 2.2. Gene Expression Analysis and Correlation Analysis

Several gene expression investigations have been performed recently and include a gene expression catalog of biological system responses to diabetes. The analysis of gene expression was used to investigate the gene regulation activity in diabetes. Three GEO datasets (GSE15932, GSE30208, and GSE55098) comprising 44 and 57 diabetic and healthy subjects, respectively, were retrieved from the NCBI-GEO database [19]. These GEO datasets were analyzed by using ImaGEO [20] software. The adjusted P-value threshold was ≤0.001 for identifying diabetes-associated gene expression profiles (Figure 1C). Correlation analysis was performed on the gene expression data of the diabetes-related genes. Pearson’s correlation [21] was calculated and plotted by using the R packages corrr0.4.4 and corrplot0.92. Correlations with *r* < 0.5 or *p*-value > 0.01 were discarded.

### 2.3. Enrichment Analysis and Protein–Protein Interactions

The gene profiles obtained from text mining and gene expression analysis were submitted to a comprehensive computational analysis, conducted by using several bioinformatics tools. Gene enrichment analysis was conducted by using ShinyGo [22], gprofiler [23], and Uniprot database [24]. Protein–protein interaction (PPI) analysis for diabetes-associated genes retrieved from text mining and GEO data analyses was conducted by using the STRING database [25] (Figure 1D). Gene expression and text mining results were represented by using ggplot2.3.3.6 [26] and GeneSyno [27] according to the human genome data. The text mining analysis provided us with a better understanding of the most well-known diabetes-related genes.

### 2.4. Machine Learning Analysis and Correlation Analyses

The results of text mining and differential gene expression analyses of potential diabetes gene biomarkers were used as attribute values for ML analysis. We extracted gene expression data from genes that were found to be significantly expressed in gene expression or were frequently mentioned in the literature (more than 50 articles). Their expression data were used for machine learning analysis as training and validation sets. The decision tree, extra-tree regressor and random forest algorithms were used in ML analysis to identify unique markers that could be used as diabetes diagnosis tools. To perform ML, we used both the R and Python programming languages. The gene expression of selected biomarkers was extracted from GEO datasets (GSE15932, GSE30208, and GSE55098). Because GSE15932 and GSE55098 (group A) share the GPL570 chip array, we were able to combine their gene expression data, whereas GSE30208 (group B) was used separately. Prior to ML analysis, gene expression data were normalised by using the calcNormFactors function in the limma 3.50.3 [28] and edgeR 3.36.0 [29] R packages via the TMM method. RandomForest4.7-1.1, rpart4.1.16, and caret6.0-93 packages in R programming languages were used to perform random forest and decision tree algorithms with 70% and 30% training and test data sets ratio, respectively. In Python, sklearn and lime0.2.0.1 packages were used to perform extra-tree regressor, and local interpretable model-agnostic explanations algorithms. The codes can be found in the github code repository via the following link: https://github.com/AlsammanAlsamman/DiabetesML, (accessed on 1 October 2022).

## 3. Results

### 3.1. Diabetes-Related Genes Occurring Frequently in the Literature

**Figure 2:**
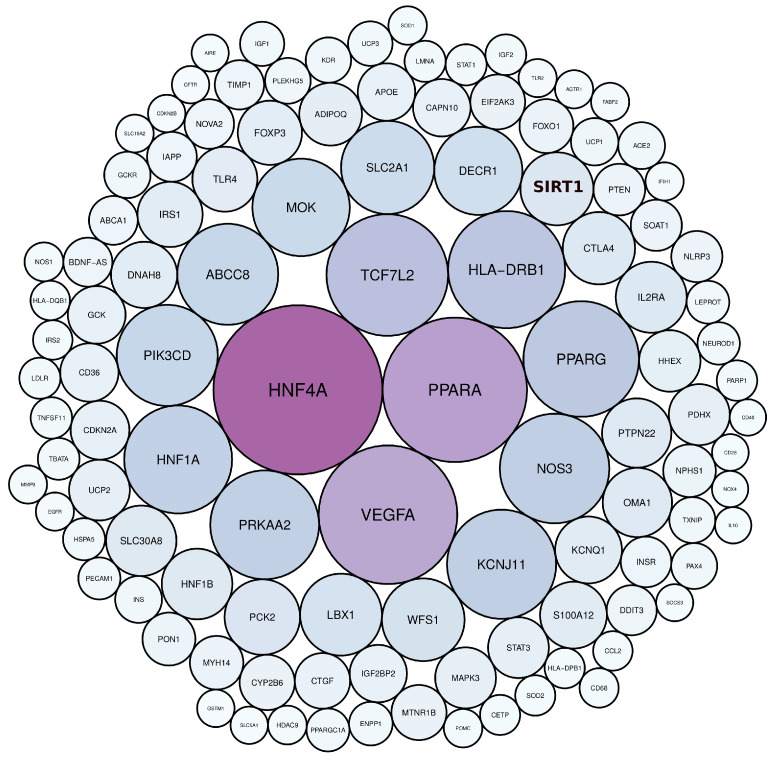
The most frequently mentioned genes in diabetes literature, as determined by text mining. The colour and size of the circles are proportional to the frequency of gene terms in diabetes literature.

**Figure 3:**
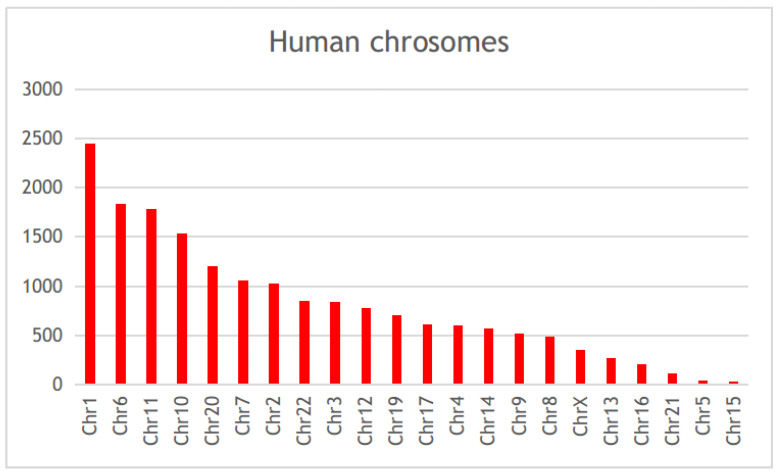
The chromosomal distribution of the diabetes-associated genes in published literature.

Scientific publications that studied the genetic factors controlling diabetes pathogenesis were screened through NCBI-pubmed, and 40,225 articles were obtained. These articles highlighted 5939 diabetes-associated genes distributed across 22 human chromosomes, of which 112 genes were mentioned in more than 50 articles (Table S1). Among these genes, *HNF4A*, *PPARA*, *VEGFA*, *TCF7L2*, *HLA-DRB1*, *PPARG*, *NOS3*, *KCNJ11*, *PRKAA2*, and *HNF1A,* were mentioned in more than 200 articles (Figure 2, Table S1). Gene distribution across the human genome showed that the largest number of genes were present in chromosomes 1, 6, 11, and 10 (Figure 3).

**Figure 4:**
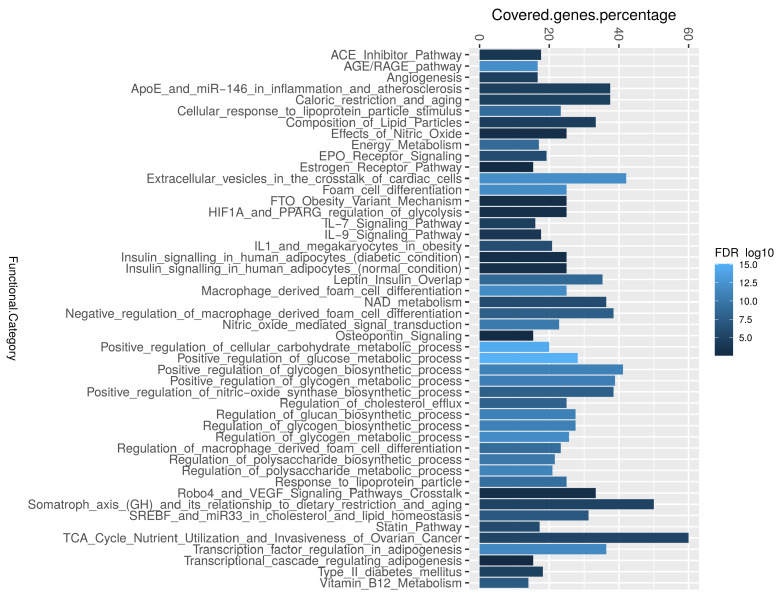
The enrichment analysis of the most common diabetes-associated genes in the literature. The most highly covered biological pathways, as determined by the ShinyGO software, where the R-ggplot was used to depict the percentage covered-genes.

Enrichment analysis was performed to categorize genes that were identified through text mining into their corresponding biological pathways. The biological pathway of insulin sensitivity was highly associated. The enrichment analysis revealed that biological pathways correlated with the regulation of glycogen and polysaccharide, extracellular vesicles in the crosstalk of cardiac cells, adipogenesis, AGE/RAGE, and macrophage differentiation were significantly associated with the studied diabetes-associated genes (Figure 4 and Table 1). We identified that 53 diabetes-associated genes are regulated by 17 miRNAs, the most significant of which are Hsa-miR-223-3p, Hsa-miR-146a-5p, and Hsa-miR-200c-3p (Table S3). Furthermore, the most important transcription activators are CEBPB, PDX1, ETS1, HIF1A, and STAT3 Table S3). These genes regulate the activity of numerous genes in the biological system [30].

**Figure 5:**
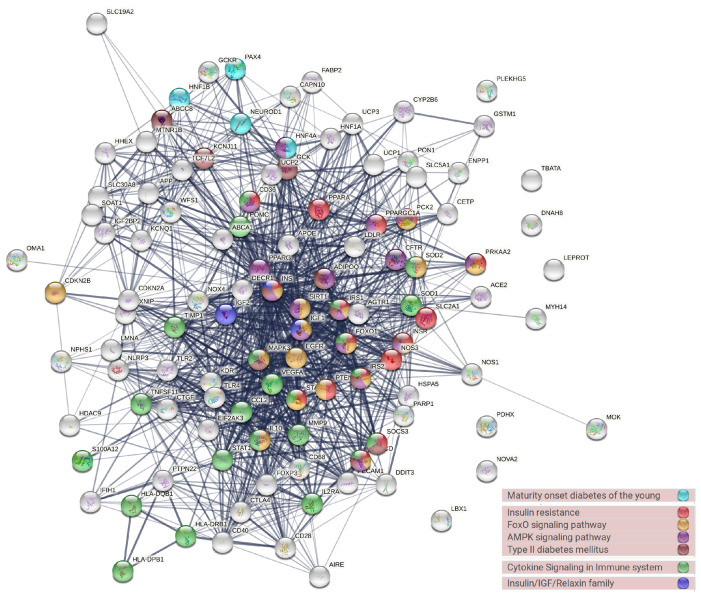
The protein–protein interaction network of the most common genes in the diabetes literature and their associated biological pathways.

The PPI analysis was conducted to evaluate the protein interaction activity and to locate the most highly interactive hub of diabetes-associated genes (Figure 5). Genes with high interaction activity were *INS*, *PPARG*, *MAPK3*, *VEGFA*, *IGF1*, *ADIPOQ*, *SIRT1*. The gene enrichment analysis (GEA) retrieved by using the STRING database revealed that most of these genes were correlated with FoxO, cytokine, and AMPK signaling, diabetes, and insulin sensitivity (Figure 5).

### 3.2. Differential Gene Expression and Correlation Analyses

**Figure 6:**
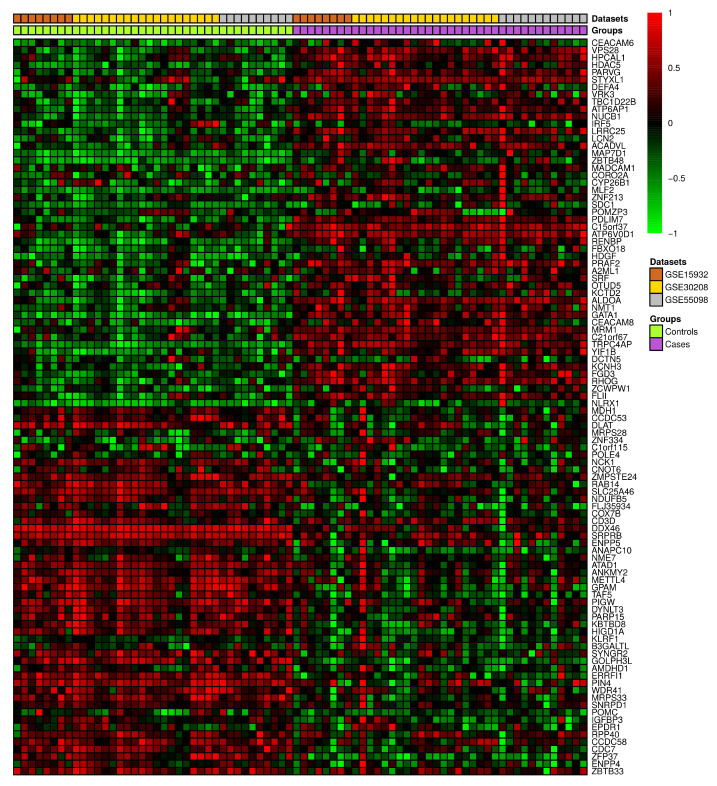
The heatmap depicting the top 100 differentially expressed genes in three previously released GEO diabetes datasets.

**Figure 7:**
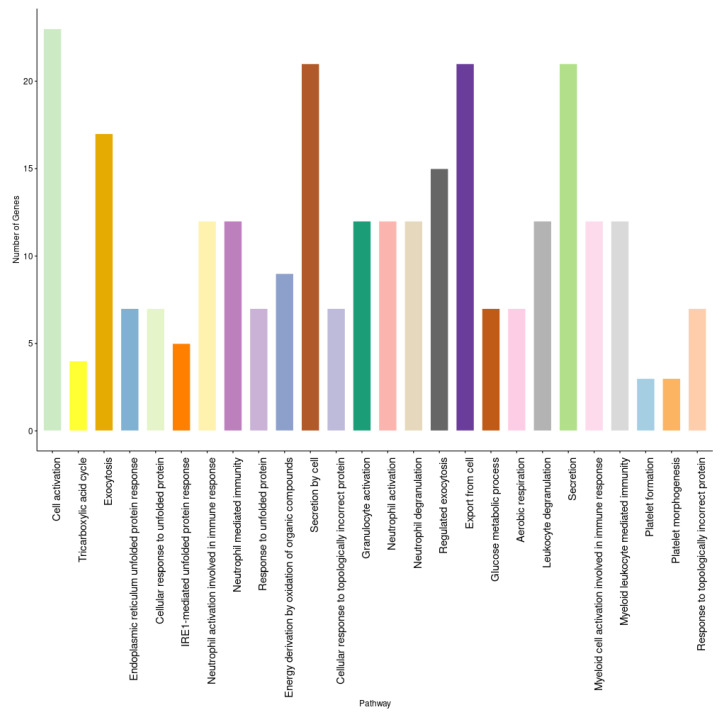
The enrichment analysis of diabetes-related genes in gene expression analysis.

A set of three GEO datasets (GSE15932, GSE30208, and GSE55098) were studied by using differential gene expression bioinformatics to classify the most important diabetes-associated genes and to determine their regulation status in healthy and diabetic individuals. The differential gene expression analysis revealed a consistent differential expression between healthy and diabetic individuals in a specific set of genes (Figure 6). The analysis revealed 135 DEGs, of which *CEACAM6*, *ENPP4*, *HDAC5*, *HPCAL1*, *PARVG*, *STYXL1*, *VPS28*, *ZBTB33*, *ZFP37*, and *CCDC58* were significantly differentially expressed (Table S2). The gene enrichment analysis revealed that a considerable number of these genes were correlated with aerobic respiration, T-cell antigen receptor (TCR) pathway, tricarboxylic acid metabolic process, vitamin D receptor pathway, toll-like receptor signaling, and endoplasmic reticulum (ER) unfolded protein response (Figure 7 and Table S3).

**Figure 8:**
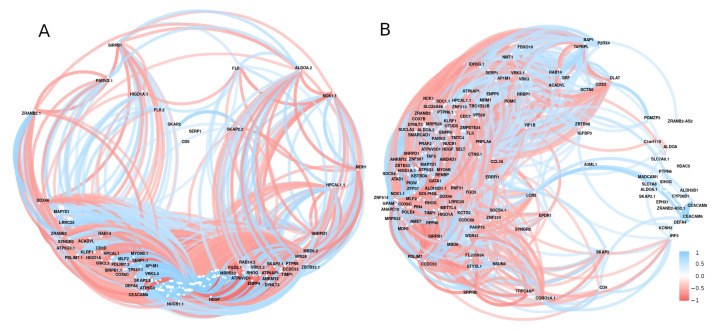
The correlation network of the expressed genes by using GSE15932 and GSE55098 (group **A**) and GSE30208 (group **B**). Positive (blue) or negative (red) correlations are indicated by links between genes. Correlations with *r* < 0.5 or with *p*-value > 0.01 were disregarded.

A hub of highly active genes was discovered during a correlation analysis of the significant genes associated with diabetes (Figure 8). Genes with a large number of correlated links to other diabetes-related genes in all study data included *NCK1*, *HIGD1A*, *VRK3*, *KBTBD8*, *ZBTB33*, *TMTC4*, *MRPS28*, *DYNLT3*, and *SMARCAD1* (Figure 8).

### 3.3. Text Mining versus Gene Expression

**Figure 9:**
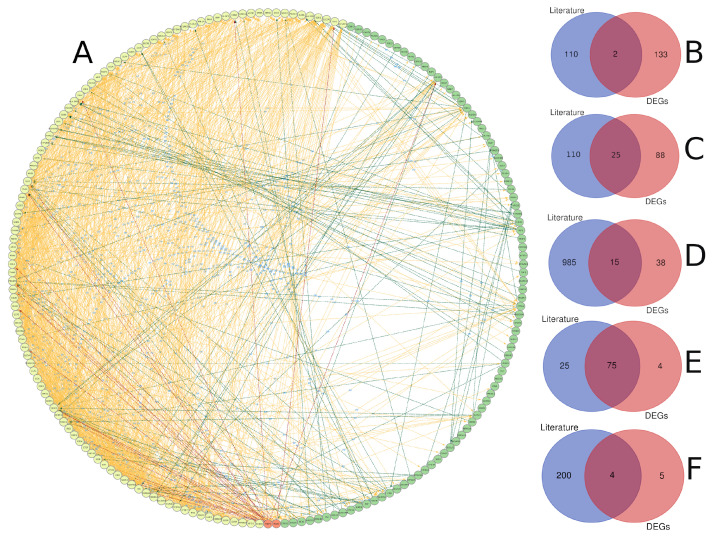
A comparison of genes linked to diabetes detected through gene expression and text mining. (**A**) Protein–protein interaction between diabetes-related genes identified through text mining (yellow), gene expression (green), or both (red), with the interaction link coloured according to the group of the interaction-source gene. The intersection of gene lists based on gene name (**B**), chromosomal location (genes with inter-space region less than 1 Mbp) (**C**), biological pathway (**D**), gene ontology (**E**), and KEGG pathway (**F**).

We compared gene lists associated with diabetes derived from text mining and gene expression analysis (Figure 9). The protein–protein interaction analysis revealed a high level of interaction for genes identified through text mining compared to gene expression analysis, which is expected given that these genes have been extensively studied in the literature and there is a plethora of data about their biological activity. Furthermore, it was found that there is some interaction between the two lists that was initiated between genes from both sides (Figure 9A). Only two genes have been shared between the two analyses, including *TIMP1* and *POMC* (Figure 9B). The small number of shared genes between the two lists could be attributed to the stringent conditions we used for gene identification in both techniques. Text mining and gene expression, on the other hand, share other genomic aspects, such as 25 chromosomal loci that contain genes from both methods. There were 47 genes from each list that are close to each other (less than 1 Mbp), and these genes are spread across 11 chromosomes, with chromosomes 1 and X having four genes each. Chromosome X included *TIMP1*, *FOXP3*, *GATA1*, *OTUD5*, and *PRAF2* genes (Figure 9C). Additionally, the two lists shared 15 biological pathways, 75 gene ontology terms, and 4 KEGG terms (Figure 9D–F). Most of the shared biological pathways were related to glucose metabolic processes, secretion, leukocyte migration, and immune response. Immune response, growth, leukocyte homeostasis, and cell motility were among the gene ontology terms shared by both lists. Citrate cycle, metabolic pathways, chemical carcinogenesis, and glycolysis were all KEGG terms that were shared.

### 3.4. Machine Learning Analysis

**Figure 10:**
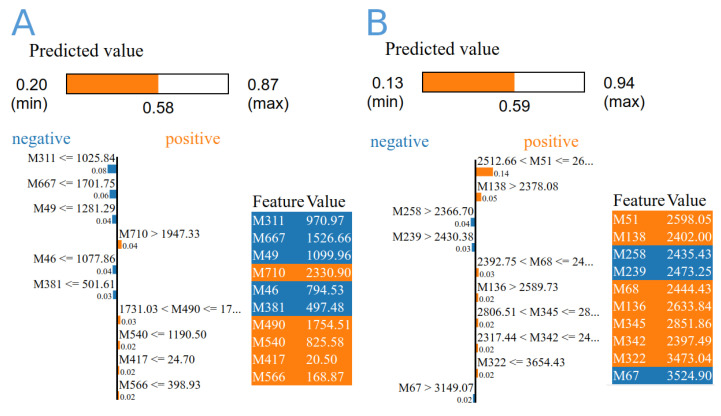
The importance score of the top 10 diabetes-related biomarkers derived from gene expression data from GSE15932, GSE55098 (**A**), and GSE30208 by using extra-tree regressor and local interpretable model-agnostic explanation algorithms (**B**). The colours represent the feature values from low to high (from blue to red).

The expression data of 1160 biomarkers associated with the expression of 274 genes were extracted, normalised, and fed into three different machine learning algorithms. By using ML and across the dataset, DecisionTree and RandomForest revealed prediction models with accuracy of 0.6364 and 0.81, and 0.7222 and 0.88, respectively, by using group A and B data. The overall confidence interval (CI) was 95%. There were 39 biomarkers linked to distinguishing diabetic and non-diabetic patients, 12 of which were repeated multiple times. These markers were linked to 36 genes, where *HLA-DQB1* was found four times (Table 2 and Figure 10). The majority of these genes are associated with crucial biological processes like response to stress, signalling regulation, locomotion, cell motility, growth, and muscle adaptation, based on the gene ontology analysis. According to decision tree algorithm 209480_at (*HLA-DQB1*), and ILMN_1720311 (*SLC25A46*) biomarkers were the most important in differentiating disease status in data A and B, respectively (Table 2). Extra-tree regressor and local interpretable model-agnostic explanation algorithms revealed that the most important biomarkers were 209342_s_at (*IKBKB*), and ILMN_1670576 (*IRF5*) groups A and B, respectively.

## 4. Discussion

In this study, a systematic methodology was followed to investigate the most common diabetes-related genes. We used three different techniques, including gene expression analysis, text-mining, and ML. Each of these techniques was useful in revealing important aspects of diabetes pathogenicity as well as important markers for early disease diagnosis.

Based on text mining, most of the highly common diabetes-related genes in the literature were correlated with glucagon and AMPK signaling pathways such as *HNF4A, PCK2*, and *SIRT1*, which were among the most interactive genes in the PPI analysis. Hepatocyte nuclear factor 4 alpha (*HNF4A*) is a highly conserved transcription factor expressed in pancreatic beta cells and required by islet beta and liver cells to maintain glucose hemostasis [31]. HNF4A crucially performs hepatic gluconeogenesis regulation and insulin secretion, and the corresponding gene was shown to be linked to type 2 diabetes (T2DM) in several studies [32]. Its loss-of-function mutations have been linked to young-onset diabetes and lipid disorders, and some mutations have been identified in several populations as risk loci for T2DM [33]. Additionally, some studies have assessed the impact of *HNF4A* gene variations on preventing and treating coronary artery disease complications. *HNF4A* gene variants may modify and modulate hepatic lipase and lipid metabolism, resulting in a beneficial effect on atherosclerosis progression and event occurrence [34]. Diabetes and coronary artery disease share many genetic key elements, owing to the fact that diabetes is considered to predispose to diabetic cardiomyopathy and atherosclerotic cardiovascular disease [35]. PCK1 and PCK2 have been proposed as potential diabetes and obesity-associated genes [36]. *PCK1* and *PCK2* are phosphoenolpyruvate carboxykinase (PCK or PEPCK) gene isoforms that are found in the cytosol and mitochondria, respectively. PEPCK is a cataplerotic enzyme which removes citric acid cycle anions for either the biosynthetic process or the subsequent complete oxidation of these substances to carbon dioxide inside the citric acid cycle [37]. *PCK* plays an important role in cell homeostasis and in cell development, including physiological processes such as glucose metabolism and the tricarboxylic acid cycle (TCA) [38]. Because insulin suppresses the expression of these enzymes, it has long been assumed that patients with T2D have increased expression of *PCK* due to hepatic insulin resistance [39,40]. Silent information regulator 1 (*SIRT1)* was the first member of the silent information regulator 2 (SIR2) family to be discovered, and it catalyzes the deacetylation of both histone and non-histone lysine residues [41]. *SIRT1* exerts its anti-oxidative effects by activating *NRF2*, a transcription factor that binds to antioxidant-responsive element genes associated with the scavenging of oxygen free radicals [42]. Recent research has shown that Sirt1 protein expression and downstream signaling were downregulated in diabetes [43]. The enrichment analysis of these genes highlighted the role of several biological pathways such as macrophage differentiation, FoxO, and adipogenesis. FoxO proteins play a significant role in mediating the impact of insulin on metabolism, including their effects on hepatic glucose production [44].

Gene expression analysis revealed a consistent differential expression between healthy and diabetic individuals in a specific set of genes. Such findings support the fact that diabetes is a multi-locus disorder with many genes controlling its pathogenesis [45]. Several genes were found to be significantly differentially expressed across diabetes gene profiles in our analysis. Most genes have been related to diabetes and cancer, with most of them being linked to pancreatic cancer (*CEACAM6, HDAC5, HPCAL1, PARVG,* and *STYXL1*). CEA cell adhesion molecule 6 (*CEACAM6)* is a key gene for pancreatic adenocarcinoma. *CEACAM6* is a cancer biomarker that regulates anoikis resistance as well as the metastatic process of pancreatic adenocarcinoma cells [46]. Histone deacetylase 5 (*HDAC5*), a key mediator of hepatic fatty acid oxidation, was identified as a major component of the fasting glucagon signalling pathway and is reported to be increased in the kidneys of diabetic patients and animals [47,48]. T2DM can cause hypothalamic-pituitary-ovarian (HPO) dysfunction, which is accompanied by increased circulating/hypothalamic HDAC5. Some findings suggest that acetate restores HPO function in T2DM by suppressing HDAC5 and increasing insulin sensitivity [49]. Furthermore, HDAC5 is involved as a common pathogenic factor in both type 1 and type 2 in vivo animal models of diabetes [49]. STYXL1 is one of three known STYX pseudophosphatases, a group of genes for which research is currently being conducted to better understand their role in disease [50]. A correlation analysis of the important genes linked to diabetes revealed a cluster of highly active genes (Figure 8). The genes with many correlated links to other diabetes-related genes included *NCK1*, *HIGD1A*, *VRK3*, *KBTBD8*, *ZBTB33*, *TMTC4*, *MRPS28*, *DYNLT3*, and *SMARCAD1* (Figure 8). Most of these genes are a part of the biological regulatory system [51,52]. Furthermore, some of these genes, such as *NCK1*, play an important role in diabetes by modifying PERK activation and signalling. *NCK1* deficiency increases pancreatic cell survival in response to diabetes-related stresses [53].

Additionally, the PPI analysis showed several highly interactive genes including nuclear-encoded mitochondrial genes, such as *MDH1,* and *NDUFB5*. Malate dehydrogenase 1 (*MDH1*) produces the human cytosolic malate dehydrogenase. This latter is vital in transporting nicotinamide adenine dinucleotide (NADH) equivalents through the mitochondrial membrane, and therefore controlling TCA cycle, which is highly linked to diabetes pathogenesis [54]. The gene-enrichment analysis of the diabetes-associated expressed genes revealed that a significant number of these genes were correlated with T-cell antigen receptor (TCR) pathway, vitamin D receptor pathway, toll-like receptor signaling, and ER unfolded protein response. The association of TCR and diabetes development has been reported in several studies, where the use of anti-TCR has been studied in the therapeutic strategy for diabetes [55]. Vitamin D deficiency increases the risk of type 1 and type 2 diabetes, and receptors for the active form of the vitamin have been found in both beta and immune cells. Protein-folding stress in the ER is a prominent feature of specialised secretory cells and has been linked to the pathogenesis of several human diseases.

The comparison of diabetes gene lists derived from text mining and gene expression analysis revealed some shared genomic aspects at different levels (Figure 9). It demonstrated that text-mining genes have a more biological interaction than gene expression analysis genes (Figure 9A). The biological relationship between the two lists may suggest the significance of both lists in presenting the variable genes involved in diabetes. We should broaden our scope to include more genes that may be important in understanding the disease structure. The two genes shared between text-mining and gene expression analyses are *TIMP1* and *POMC* (Figure 9B). TIMP metallopeptidase inhibitor 1 (*TIMP1*) is a naturally occurring inhibitor of matrix metalloproteinases (MMPs), a class of peptidases involved in the degradation of extracellular matrix. *TIMP1* levels were significantly higher in the serum of T2DM patients, and raises the possibility that it plays a role in T2DM bone fragility [56]. Proopiomelanocortin (*POMC*) encodes a preproprotein that undergoes tissue-specific post-translational processes. Increases in food consumption and body weight can result from POMC mutations. White adipose tissue undergoes a phenotypic switch in response to weight gain and obesity, which causes it to release proinflammatory cytokines that contribute to the emergence of insulin resistance and type 2 diabetes [57]. The significance of specific loci, including those on chromosome X, was brought to light by examining the genomic locations of genes derived from the two methods. Previous reports have emphasised the connection between the pathogenesis of diabetes and genes on chromosome X [58,59]. There were several of these genes, including *TIMP1*, *FOXP3*, *GATA1*, *OTUD5*, and *PRAF2* (Figure 9C). Some of these genes are known to be correlated with sex and age, such as *TIMP1* [60], and *FOXP3* [61]. The two methods shared many expected KEGG terms, biological pathways, and gene ontology terms, including citrate cycle [62], leukocyte migration [63] and other pathways with a known association with diabetes.

We chose the expression of a few specific genes in the datasets under study by using text mining, and we used well-known machine learning techniques on the selected data to find biomarkers that distinguish between the two disease states. A significant proportion of the potential biomarkers were linked to *HLA-DQB1* Table 2 and Figure 10. The *HLA-DQB1* gene belongs to a group of genes known as the human leukocyte antigen (HLA) complex. This group of genes is a major component of familial clustering in both type 1 diabetes and celiac disease, where subjects carrying specific mutations in this group are at a high risk of developing T1D [64]. Recently, the tenth article highlights the importance of *HLA-DQB1* in diabetes and suggests its function in this disease [65,66,67]. Furthermore, machine learning highlighted the significance of biomarkers associated with (*SLC25A46*), (*IKBKB*), and (*IRF5*) Table 2. *SLC25A46* is a mitochondrial carrier protein that is found in the outer mitochondrial membrane and is the closest human homolog to a yeast protein involved in mitochondrial fusion [68]. The detailed function of SLC25A46 is still unknown, and it may facilitate transport across the mitochondrial membrane or act as a molecular adaptor protein [69]. *SLC25A46* loss can cause neurodegeneration in mice by affecting mitochondrial dynamics and energy production [70]. Several studies have recently suggested a link between it and diabetes phenotypes in mice and humans [71,72]. Both *IKBKB* and *IRF5* function in immune response, apoptosis, and toll-like receptor signalling pathways. In type 2 diabetes and obesity, IRFs play a crucial role as metabolic transcriptional regulators. The polarisation of macrophages toward the inflammatory M1-phenotype has been associated with *IRF5*. In line with the inflammatory signatures, the increased *IRF5* expression in the adipose tissue of diabetic obese patients has been suggested as a potential marker for metabolic inflammation in obesity/T2D [73]. *IKBKB* is a crucial upstream modulator of the NF-kB pathway and a pro-inflammatory response regulator. When it is inhibited, lipopolysaccharide-induced inflammation and the production of pro-inflammatory cytokines are reduced [74]. *IKBKB* was discovered to play a role in the development of T2DM, and studies have shown that its deletion inhibited the production of inflammatory cytokines that increase insulin resistance [75,76]. These findings suggest that the machine learning analysis was essential for broadening viewpoints, enabling the observation of some hidden figures in genes related to diabetes, and aiding in the improvement of text-mining and gene expression analysis results.

## 5. Conclusions

Three different bioinformatics approaches were used to identify genes that are strongly linked to diabetes pathogenesis. Every one of these methods show different aspects of the gene structure of diabetes. The 40,225 abstracts of diabetes articles published show that there are only a few genes that are highly concerned by the medical community. These genes contain a wealth of information regarding their molecular function, PPI, and gene ontology. Among these genes, *HNF4A*, *PPARA*, *VEGFA*, *TCF7L2*, *HLA-DRB1*, *PPARG*, *NOS3*, *KCNJ11*, *PRKAA2*, and *HNF1A* were mentioned in more than 200 articles. This could imply that the number of genes that have been extensively studied and may have a positive impact on our understanding of the diabetes gene network is decreasing. Three different diabetes gene expression datasets were studied by using gene expression analysis. The analysis revealed 135 significant DEGs, of which *CEACAM6*, *ENPP4*, *HDAC5*, *HPCAL1*, *PARVG*, *STYXL1*, *VPS28*, *ZBTB33*, *ZFP37*, and *CCDC58* were the top ten DEGs. The TCR pathway, the vitamin D receptor pathway, and the ER-unfolded protein response were all enriched in these genes, which were linked to the development of diabetes. Machine learning analysis provided innovative strategies for ranking the significance and potential utility of genes related to diabetes as biomarkers. ML algorithms highlighted the use of the *HLA-DQB1* gene as a biomarker for diabetes early detection and provided several prediction models with moderate accuracy. A number of prediction models with fair accuracy were provided by ML algorithms, which also highlighted the use of the *HLA-DQB1* gene as a biomarker for diabetes early detection. Our research offers fresh information on the crucial genes and metabolic processes involved in diabetes, which could be used to identify potential research targets in the future.

## Figures and Tables

**Table 1:** The analysis of gene enrichment of the most frequently found diabetes-related genes in the literature (-log-pvalue ≥ 16). The biological pathways with false discovery rate (FDR). The intersection ratio represents how many of the termed known genes are found within the studied list, and the share ratio how many of the genes belong to this biological pathway.

Term Name	FDR	Share	Intersection	Term Name	FDR	Share	Intersection
response to nitrogen compound	33.3	47.27%	4.73%	positive regulation of macromolecule metabolic process	19.3	58.18%	1.85%
response to organonitrogen compound	32.6	45.45%	4.95%	positive regulation of multicellular organismal process	19.3	40.00%	3.07%
regulation of multicellular organismal process	30.9	62.73%	2.59%	apoptotic process	19.2	44.55%	2.63%
cellular response to chemical stimulus	30.6	65.45%	2.38%	localization	19.1	76.36%	1.31%
response to endogenous stimulus	28	49.09%	3.47%	signaling	19.1	76.36%	1.30%
chemical homeostasis	27.6	41.82%	4.50%	cell death	19	46.36%	2.46%
regulation of biological quality	27.2	68.18%	1.99%	positive regulation of cell communication	19	42.73%	2.75%
positive regulation of biological process	27	82.73%	1.46%	cellular response to peptide	18.9	23.64%	7.45%
regulation of cell communication	26.7	64.55%	2.13%	positive regulation of signaling	18.9	42.73%	2.74%
cellular response to organic substance	26.5	56.36%	2.59%	regulation of cell differentiation	18.9	40.91%	2.90%
cellular response to oxygen-containing compound	26.2	42.73%	4.03%	programmed cell death	18.7	44.55%	2.55%
positive regulation of cellular process	24.1	77.27%	1.49%	hormone secretion	18.4	21.82%	8.39%
response to peptide hormone	24	28.18%	7.79%	negative regulation of cellular process	18.2	66.36%	1.50%
glucose homeostasis	23.2	23.64%	10.83%	hormone transport	18.1	21.82%	8.14%
regulation of developmental process	23.2	53.64%	2.43%	small molecule metabolic process	18.1	42.73%	2.62%
carbohydrate homeostasis	23.1	23.64%	10.79%	regulation of molecular function	18	53.64%	1.93%
multicellular organismal process	23	84.55%	1.26%	Late onset	17.9	23.38%	41.86%
positive regulation of metabolic process	22.4	63.64%	1.85%	positive regulation of biosynthetic process	17.9	44.55%	2.45%
regulation of response to stimulus	22.4	64.55%	1.82%	regulation of phosphate metabolic process	17.9	38.18%	3.03%
Abnormal waist to hip ratio	22	24.68%	54.29%	regulation of phosphorus metabolic process	17.9	38.18%	3.03%
Increased waist to hip ratio	22	24.68%	54.29%	regulation of response to stress	17.8	37.27%	3.13%
response to insulin	21.1	22.73%	9.88%	regulation of hormone secretion	17.6	20.00%	9.32%
response to external stimulus	20.9	54.55%	2.15%	macromolecule localization	17.4	53.64%	1.89%
developmental process	20.6	77.27%	1.35%	regulation of intracellular signal transduction	17.3	40.91%	2.66%
cellular developmental process	20.5	64.55%	1.70%	cell surface receptor signaling pathway	17.2	50.91%	1.99%
regulation of cell population proliferation	20.5	43.64%	2.90%	positive regulation of cellular metabolic process	17.2	54.55%	1.83%
regulation of signal transduction	20.5	55.45%	2.07%	negative regulation of multicellular organismal process	17	32.73%	3.60%
regulation of apoptotic process	20.4	40.91%	3.17%	intracellular signal transduction	16.9	49.09%	2.05%
cellular response to nitrogen compound	20.3	30.91%	4.98%	cellular response to endogenous stimulus	16.8	36.36%	3.04%
cellular response to organonitrogen compound	20.3	30.00%	5.27%	anatomical structure development	16.7	70.00%	1.34%
regulation of cell death	20.3	42.73%	2.95%	organic substance transport	16.7	49.09%	2.03%
cell population proliferation	20.2	46.36%	2.60%	protein secretion	16.7	21.82%	7.08%
regulation of programmed cell death	20.1	40.91%	3.11%	establishment of protein localization to extracellular region	16.6	21.82%	7.06%
Insulin resistance	20	29.87%	29.87%	multicellular organismal homeostasis	16.5	25.45%	5.23%
cellular response to stimulus	19.9	81.82%	1.22%	positive regulation of cellular biosynthetic process	16.5	42.73%	2.39%
animal organ development	19.8	59.09%	1.85%	protein localization to extracellular region	16.4	21.82%	6.92%
cell differentiation	19.8	63.64%	1.68%	regulation of small molecule metabolic process	16.4	21.82%	6.92%
regulation of transport	19.8	43.64%	2.78%	regulation of protein localization	16.3	30.00%	3.91%
cell communication	19.7	77.27%	1.31%	multicellular organism development	16.2	63.64%	1.47%
response to hormone	19.6	32.73%	4.29%	negative regulation of biological process	16.1	70.00%	1.31%

**Table 2:** The most significant gene expression biomarkers associated with diabetes that were found by using various machine learning techniques and the gene expression data of GSE15932, GSE55098 (A), and GSE30208 (B).

ML Algorithm	Data	Marker Code	Marker Name	Importantance	Gene
DecisionTree	A	M313	209480_at	8.54	HLA-DQB1
		M399	212999_x_at	6.83	HLA-DQB1
		M398	212998_x_at	5.98	HLA-DQB1
		M710	238996_x_at	5.98	ALDOA
		M370	211654_x_at	5.12	HLA-DQB1
		M417	214631_at	5.12	ZBTB33
	B	M148	ILMN_1720311	13.07	SLC25A46
		M302	ILMN_1790797	9.44	VPS28
		M61	ILMN_1672899	9.44	POMC
		M161	ILMN_1726470	7.99	OTUD5
		M41	ILMN_1666192	7.99	DCTN5
		M88	ILMN_1684802	7.99	TAF5
RandomForest	A	M667	233510_s_at	0.53	PARVG
		M710	238996_x_at	0.41	ALDOA
		M313	209480_at	0.40	HLA-DQB1
		M546	223016_x_at	0.25	ZRANB2
		M203	205025_at	0.19	ZBTB48
		M141	202462_s_at	0.18	DDX46
		M399	212999_x_at	0.16	HLA-DQB1
		M636	230031_at	0.15	HSPA5
		M140	202455_at	0.15	HDAC5
		M80	1569150_x_at	0.15	PDLIM7
	B	M51	ILMN_1670576	2.08	IRF5
		M41	ILMN_1666192	1.97	DCTN5
		M148	ILMN_1720311	1.68	SLC25A46
		M345	ILMN_1813746	1.19	CORO2A
		M333	ILMN_1806408	1.00	ACADVL
		M61	ILMN_1672899	0.86	POMC
		M146	ILMN_1718822	0.82	STYXL1
		M239	ILMN_1762095	0.81	TMTC4
		M136	ILMN_1709800	0.64	POMZP3
		M265	ILMN_1771697	0.52	VRK3

## Data Availability

All data generated or analysed during this study are included in this published article and are available at: https://doi.org/10.5281/zenodo.7194230, accessed on 17 October 2022.

## Supplementary Materials

The following supporting information can be downloaded at: https://www.mdpi.com/article/10.3390/ijerph192113890/s1, Table S1: The frequency of genes in diabetes literature as revealed by text mining analysis; Table S2: The results of a gene expression analysis that revealed genes linked to diabetes; Table S3: The results of the gene enrichment analysis of genes associated with diabetes.

## Abbreviations

The following abbreviations are used in this manuscript:

DEG	Differential expressed genes
ER	Endoplasmic reticulum
GEO	Gene Expression Omnibus
IDF	International Diabetes Federation
PPI	Protein-protein interaction
TCA	Tricarboxylic acid
TCR	T-Cell antigen Receptor
